# OrientationNN: a physics-informed lightweight neural network for real-time joint kinematics estimation from IMU data

**DOI:** 10.3389/fbioe.2025.1737916

**Published:** 2026-01-12

**Authors:** Qingyao Bian, Hongbo Wang, Khalid Alsayed, Ziyun Ding

**Affiliations:** 1 School of Engineering, University of Birmingham, Birmingham, United Kingdom; 2 School of Automation Engineering, Nanjing University of Information Science and Technology, Nanjing, China; 3 Faculty of Medical Rehabilitation Science, King Abdulaziz University, Jeddah, Saudi Arabia

**Keywords:** biomechanics, IMU, joint kinematics estimation, lightweight, physics-informed neural network

## Abstract

**Introduction:**

Accurate joint kinematics estimation is essential for understanding human movement and supporting biomechanical applications. Although optical motion capture systems are accurate, their high cost, complex setup, and limited portability restrict use outside laboratory environments. This study proposes a lightweight, physics-informed neural network for real-time joint kinematics estimation using inertial measurement units (IMUs).

**Methods:**

We developed OrientationNN, which integrates orientation-based physical constraints into a compact multi-layer perceptron architecture to ensure biomechanically consistent joint kinematics estimation. The model was evaluated on a publicly available dataset and compared with a physics-based inverse kinematics framework (OpenSense) and conventional learning-based models including MLP, LSTM, CNN, and Transformer.

**Results:**

OrientationNN achieved an average joint angle estimation error below 5° during ambulatory motion and consistently outperformed OpenSense across all kinematic variables. The model required only 4.9 × 10³ FLOPs per frame and 10.8 KB of parameters, demonstrating high computational efficiency suitable for real-time applications.

**Conclusion:**

OrientationNN enables accurate and computationally efficient joint kinematics estimation from IMU data. The results highlight its potential as a cost-effective and scalable solution for wearable biomechanical and motion analysis applications.

## 1 Introduction

Accurate estimation of joint kinematics is fundamental to understanding human movement, advancing biomechanical modelling, and supporting the design, assessment, and optimisation of interventions and assistive technologies. When achieved in real time, such estimation enables personalised rehabilitation therapy (Patel et al., 2012) and closed-loop control of powered prostheses and exoskeletons (Kawamoto et al., 2003), contributing to objective management of motor recovery and functional performance (Baker, 2006). While optical motion capture systems remain the gold standard for laboratory-based motion analysis, their reliance on controlled environments, expensive infrastructure, and complex calibration procedures severely limits their applicability in clinical settings and daily-living scenarios (Cappozzo et al., 2005). This gap between laboratory precision and real-world accessibility has motivated the development of wearable sensing technologies for continuous motion monitoring.

Inertial measurement units (IMUs) have emerged as a promising alternative, offering compact form factors, low cost, and environmental independence (Roetenberg et al., 2009). Recent studies have demonstrated that IMU-based systems can achieve accuracies comparable to optical systems for gait analysis in controlled settings (Cutti et al., 2010; Al-Amri et al., 2018), with comparative validation studies showing strong agreement between inertial sensor-based and camera-based measurements during treadmill walking and running (Nüesch et al., 2017), and confirming concurrent validity and within-session reliability when proper calibration protocols are applied (Berner et al., 2020). However, translating IMU-derived orientations into anatomically meaningful joint angles remains a challenging inverse problem, particularly when sensor-to-segment misalignment, soft tissue artifacts, and calibration errors are present (Cooper et al., 2009).

Two primary paradigms have been developed to address this challenge: physics-based methods and data-driven machine learning approaches. Physics-based methods, such as OpenSense (Delp et al., 2007) and Xsens MVN (Roetenberg et al., 2009), employ biomechanical models combined with inverse kinematics optimisation to reconstruct joint angles from IMU orientations. By incorporating anatomical constraints — including bone-to-bone articulation, segmental parameter consistency, and physiologically valid joint ranges of motion — these methods ensure biomechanical plausibility. However, they require iterative numerical optimisation, which incurs substantial computational cost (often $>10^{6}$ FLOPs per frame) (Seth et al., 2018). In addition to modelling errors, inaccuracies may also arise from IMU-related factors, including sensor characteristics, calibration errors, sensor placement variability, and soft tissue artefacts (Seel et al., 2014). Recent validation studies report root-mean-square errors (RMSEs) between 5 ° and 10 ° for walking tasks (Delp et al., 2007; Kok et al., 2017). However, such accuracy may be inadequate for clinical applications, including the physical assessment of disease severity, where the minimum detectable change is typically less than 5 ° (Peters et al., 2021).

In contrast, data-driven machine learning approaches have demonstrated superior performance by learning direct mappings from IMU data to joint kinematics without explicit modelling assumptions. Recent advances include convolutional neural networks (CNNs) for spatial feature extraction (Huang et al., 2018; Yi et al., 2022), long short-term memory (LSTM) networks for temporal dependency modelling (Mundt et al., 2020; Khant et al., 2023), attention-based architectures for adaptive feature weighting (Wang et al., 2020), and transformer models for global sequence modelling (Kwon et al., 2020). These methods have achieved RMSEs below 3° in controlled settings (Mundt et al., 2020; Lea et al., 2017; Kwon et al., 2020), outperforming physics-based approaches across walking, running, and stair ambulation tasks. Furthermore, hybrid sensor fusion approaches combining IMUs with surface electromyography (sEMG) have shown promise for capturing neuromuscular dynamics (Phinyomark et al., 2018; Chen et al., 2018).

Despite these impressive results, purely data-driven models face critical limitations that hinder their translation to clinical practice and wearable deployment. First, they lack physical interpretability–operating as black boxes without guarantees of biomechanical consistency, which raises concerns for safety-critical applications such as prosthetic control (Tucker et al., 2020). Second, state-of-the-art architectures such as transformers demand substantial computational resources ( $>10^{6}$ FLOPs per frame and hundreds of kilobytes (KB) of parameters) (Kwon et al., 2020), rendering them impractical for real-time inference on resource-constrained edge devices such as microcontroller units (MCUs) embedded in wearable systems, where power budgets are typically limited to milliwatts (Lane et al., 2015; Reddi et al., 2020). Finally, their dependence on large labelled datasets obtained from expensive motion capture systems limits scalability and accessibility for clinical researchers.

In recent years, physics-informed neural networks (PINNs) have emerged as a framework that integrates physical laws with neural networks (Raissi et al., 2019; Karniadakis et al., 2021). By embedding physical laws, such as Newton-Euler equations, kinematic constraints, or conservation principles, into neural network architectures or loss functions, PINNs achieve improved data efficiency and physical consistency (Cuomo et al., 2022). Recent applications in fluid dynamics (Raissi et al., 2020), structural mechanics (Zhang et al., 2022), and robotics (Westenbroek et al., 2022) have demonstrated that physics-informed models can match or exceed purely data-driven approaches with orders of magnitude fewer parameters. However, despite growing interest in biomechanics applications (Linka et al., 2021), PINNs have rarely been applied to IMU-based joint kinematics estimation, and no existing work has systematically addressed the computational efficiency requirements for edge deployment in wearable systems.

To address these gaps, this study introduces OrientationNN, a lightweight physics-informed neural network that integrates orientation-based kinematic constraints with compact multi-layer perceptron (MLP) subnetworks for real-time lower limb joint angle estimation from IMU data. Unlike purely data-driven models that learn arbitrary input-output mappings, OrientationNN explicitly encodes the rotational relationships between adjacent body segments using learnable rotation matrices that represent sensor-to-segment calibrations, combined with dynamic MLP modules that capture subject-specific non-rigid motion artifacts. This hybrid architecture preserves biomechanical interpretability while achieving computational efficiency through modular, joint-specific processing with minimal parameter overhead.

Our research objectives are:

1. To develop a lightweight physics-informed neural network (OrientationNN) that integrates orientation-based physical constraints with a compact MLP architecture for accurate (RMSEs below 5°) and efficient (model size under 20 KB) estimation of lower-limb joint kinematics from IMU data.
2. To analyse and compare the error distribution of the proposed OrientationNN and the physics-based biomechanics model (i.e., OpenSense, an IMU-driven inverse kinematics toolbox), providing insights into model behavior and optimisation throughout the gait cycle.
3. To ensure efficient, real-time, and edge-deployable kinematics estimation by reducing computational cost and parameter dependency.

## 2 Methods

### 2.1 Problem statement

Our objective was to provide a high-accuracy and real-time automated estimation of lower limb joint kinematics using wearable IMU sensors.

We used a publicly available treadmill walking dataset comprising recordings from 14 healthy adults (7 males and 7 females), each conducting five approximately 7-min walking trials at a self-selected speed (Bailey et al., 2021). The dataset included measurements from eight IMU sensors (Xsens) mounted on the trunk, lower back, left and right thighs, left and right shanks, and left and right feet. In this study, the trunk IMU was excluded from modelling by following the previous work (Ma et al., 2025). The signals were sampled at 60 Hz, resulting in around 25000 data points per 7-min trial, with variations depending on trial durations.

**FIGURE 1:**
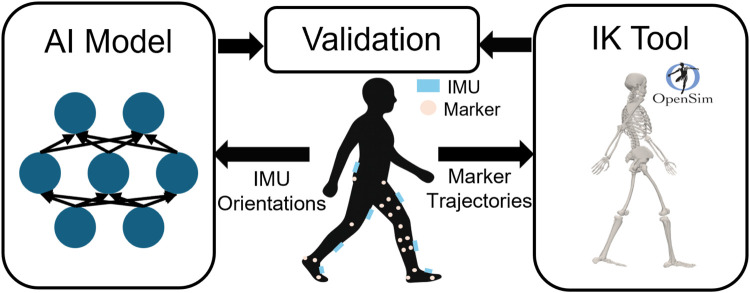
Schematic of the experimental setup and data acquisition system. IMU sensors were attached to the lower back, thighs, shanks, and feet to record lower limb kinematics, while a marker-based optical motion capture system was used to obtain the ground truth joint angles for model evaluation.

In addition, the dataset provides lower limb joint angles computed through OpenSim’s inverse kinematics (IK) based on marker trajectory data. As OpenSim is a validated and widely accepted framework for estimating joint kinematics (Holzbaur et al., 2005), the OpenSim-derived joint angles were used as the reference standard for evaluating the proposed neural network model (Figure 1).

Following data quality screening based on inter-system synchronisation accuracy, recordings with synchronization errors exceeding 50 ms were excluded. As a result, recordings from ten participants were retained for further analysis. Both IMU-driven (OpenSense) and optoelectronic-driven (OpenSim) inverse kinematics solutions were used for model comparison. The IMU data were synchronised with the marker-based data in MATLAB to eliminate signal delay.

Lower limb joint kinematics, including hip flexion, hip adduction, hip rotation, knee flexion, ankle dorsiflexion, and ankle inversion, were estimated. Neural network models were trained and evaluated under an intra-subject scenario, in which model training and testing were performed using data from the same individual. For each participant, the dataset was partitioned into training (60%), validation (20%), and test (20%) subsets. The training data were used to fit the model, the validation data to optimise the network architecture and hyperparameters, and the test data to evaluate final performance.

### 2.2 OrientationNN

#### 2.2.1 Model summary

**FIGURE 2:**
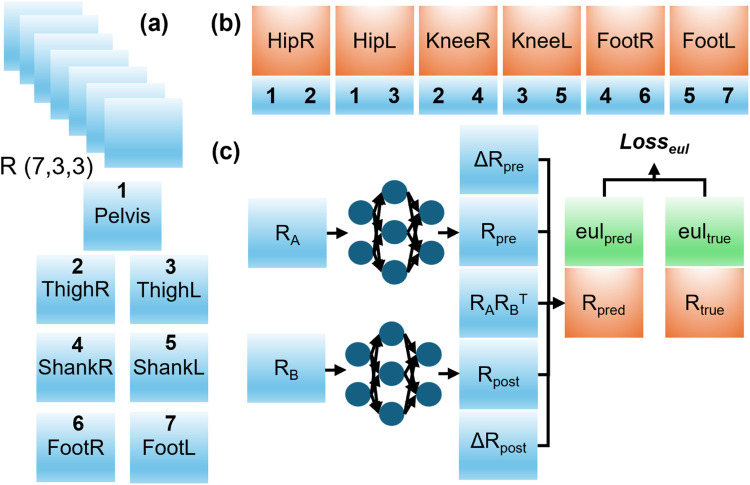
OrientationNN architecture. **(a)** Model input. **(b)** Grouping of adjacent IMU orientations for joint kinematics estimation. **(c)** Computation flow from relative rotation matrices to Euler angles.

We proposed OrientationNN, a lightweight architecture that integrates orientation-based physical information with compact multi-layer perceptrons (MLPs), ensuring computational efficiency and adherence to segmental constraints. The modelling process began by grouping adjacent IMU orientations as shown in Figure 2b to calculate the corresponding joint angles. The model takes the orientations of two adjacent segments as input and estimates their relative rotation matrix, representing the corresponding joint motion. (Figure 2c). These joint motion matrices were converted into Euler angles to compute the loss function, as segmental kinematics are conventionally represented using Euler angles.

The proposed model incorporates joint-specific dynamic modules. Each joint employs a tiny MLP subnetwork (input = 3, hidden = 32, output = 3) to predict dynamic offsets $R_{\mathrm{pre}}$ and $R_{\mathrm{post}}$ , which are combined with learnable static parameters ( $\Delta R_{\mathrm{pre}}$ , $\Delta R_{\mathrm{post}}$ ) to form rotation matrix outputs, as described in the last section. Training is conducted for 100 epochs using the Adam optimizer with a learning rate of 0.005 (Kingma and Ba, 2014). The weighted EulerXYZ loss is selected as the loss function to enhance biomechanical consistency and joint-specific precision (Chen et al., 2011).

#### 2.2.2 Basic module

Assume that we have two adjacent segment orientation $R_{A}$ and $R_{B}$ , then the joint rotation matrix could be calculated as shown in Equation 1:

$$R_{\mathrm{joint}}=R_{A}R_{B}^{\prime }$$ (1)

where $R_{\mathrm{joint}}$ is the relative joint rotation matrix which corresponds to $R_{A}$ and $R_{B}$ .

#### 2.2.3 Learnable static module

However, only IMU orientations are measured directly in the study. Thus, we added two learnable static rotation matrices to replace the sensor to segment orientations (Mundt et al., 2020). Then the joint rotation matrix can be calculated as shown in Equation 2:

$$R_{\mathrm{joint}}=\Delta R_{\mathrm{pre}}R_{A}R_{B}^{\prime }\Delta R_{\mathrm{post}}$$ (2)

where $\Delta R_{\mathrm{pre}}$ and $\Delta R_{\mathrm{post}}$ are learnable rotation matrix to function as sensor-to-segment orientations.

#### 2.2.4 Dynamic module

To further help the model approximate the joint motion better, we also introduced an extra dynamic module containing tiny MLP models. Two dynamics orientation matrices were first calculated as shown in Equations 3, 4:

$$R_{\mathrm{pre}}=\text{Exp}\left(\text{MLP}\left(\text{Log}\left(R_{A}\right)\right)\right)$$ (3)

$$R_{\mathrm{post}}=\text{Exp}\left(\text{MLP}\left(\text{Log}\left(R_{B}\right)\right)\right)$$ (4)

where $R_{\mathrm{pre}}$ and $R_{\mathrm{post}}$ are dynamics rotation matrices, Exp(.) is the function mapping SO (3) to so (3), and Log(.) is the function mapping so (3) to SO (3) (Solà et al., 2018). SO(3) is the special orthogonal group of 3D rotation matrices, while so(3) denotes its corresponding Lie algebra of 3D rotation vectors.

#### 2.2.5 Concatenation module

Then, the joint angles can be calculated as shown in Equations 5, 6:

$$R_{\mathrm{joint}}=R_{\mathrm{pre}}\Delta R_{\mathrm{pre}}R_{A}R_{B}^{\prime }\Delta R_{\mathrm{post}}R_{\mathrm{post}}$$ (5)

$$eul=\text{rot}2\text{eul}\left(R_{\mathrm{joint}}\right)$$ (6)

where eul are Euler angles of the corresponding joint, rot2eul(.) is the function which converts a rotation matrix into Euler angles following the Z-Y-X sequence. This corresponds to the rotation convention adopted by OpenSim, ensuring a fair comparison between models Delp et al. (2007).

#### 2.2.6 Weighted euler loss

The loss function is calculated as shown in Equation 7:

$$L=\overset{12}{\underset{j=1}{\sum }}w_{j}eul_{j}$$ (7)

where $w_{j}$ is the weight of the $j_{th}$ channel, which was determined by motion range.

### 2.3 Baseline machine learning models

For comparison with our proposed model OrientationNN, we adopted multiple neural network models from literature. The model architectures and hyperparameters are optimised using a framework named Optuna (Akiba et al., 2019), which adopted a Bayesian method to get the optimal architecture and hyper parameter for each model. The optimal hyperparameters of other machine learning models are listed in Table 1.

**TABLE 1:** Model architectures and training hyperparameters.

Model	Architecture	Learning rate	Epochs	Optimizer	Loss
MLP	2 × 128 fully connected layers (ReLU + dropout 0.2)	0.01	100	Adam	L1 loss
LSTM	1-Layer LSTM (dropout 0.2) + linear	0.01	100	Adam	L1 loss
CNN	Conv2d ( k=3 , pad =1 ) + ReLU + Dropout (0.2) + flatten + linear	0.01	100	Adam	Huber loss
Transformer	Linear +3 × transformer EncoderLayer ( $d_{\text{model}}=64$ , $n_{\text{head}}=2$ , $d_{\text{ff}}=256$ , dropout =0.1 ) + flatten + linear	0.005	100	Adam	L1 loss
OrientationNN	Per-joint MLP (hidden =32 ) + learnable orientations	0.005	100	Adam	Euler loss

#### 2.3.1 MLP

The MLP network comprises two fully connected hidden layers, each containing 128 neurons with rectified linear unit (ReLU) activation functions. To prevent overfitting, a dropout layer with a rate of 0.2 is applied after each hidden layer. The input layer receives 63 features, and the output layer produces 12 joint angle estimations. Model parameters are optimized using the Adam optimizer with a learning rate of 0.01 and a weight decay of $1\times 10^{-4}$ . The network is trained for 100 epochs with the mean absolute error (L1Loss) as the objective function.

#### 2.3.2 LSTM

The LSTM model consists of a single recurrent layer with 128 hidden units and an input size of 63. A dropout rate of 0.2 is applied to the LSTM outputs to reduce overfitting. The final fully connected layer maps the 128-dimensional hidden representation to 12 output joint angles. Training is performed for 100 epochs using the Adam optimizer (learning rate = 0.01, weight decay = $1\times 10^{-4}$ ) and the L1 Loss function (MAE).

#### 2.3.3 CNN

The CNN model performs spatiotemporal feature extraction using a two-dimensional convolutional layer (Conv2D) with 64 filters and a kernel size of 3, followed by ReLU activation and a dropout rate of 0.2. The feature maps are then flattened and passed through a fully connected layer that outputs 12 joint angle predictions. The input tensor has dimensions of 7×3×3 , corresponding to seven IMUs with three-axis features. Model training uses the Adam optimizer (learning rate = 0.01, weight decay = $1\times 10^{-4}$ ) for 100 epochs with the Huber Loss function.

#### 2.3.4 Transformer

The Transformer-based model begins with a linear projection layer that maps the input to a 64-dimensional embedding space. This embedding is processed by three Transformer encoder layers with a model dimension of 64, two attention heads, a feedforward dimension of 256, and a dropout rate of 0.1. The output sequence is flattened and passed through a final linear layer to produce 12 joint angle outputs. Training is performed for 100 epochs using the Adam optimizer (learning rate = 0.005, weight decay = $1\times 10^{-4}$ ) and L1 Loss (MAE).

### 2.4 Model evaluation

#### 2.4.1 Performance metrics

Model accuracy was assessed by using the root mean squared error (RMSE), which is defined as follows: $\text{RMSE}=\sqrt{\frac{1}{n}\sum_{i=1}^{n}{\left(y_{i}-\hat{y_{i}}\right)}^{2}}$ , where $\bar{y}$ is the mean of the actual values. To evaluate the model’s suitability for edge deployment, we measured FLOPs and memory usage, using an NVIDIA RTX 3050 laptop GPU, which approximates the performance of widely used edge devices. Furthermore, to enable real-time joint kinematics estimation for feedback and to provide reliable input for assistive device control, several specific performance criteria were defined:

1. The estimation of joint angles should achieve a RMSE of less than 5 ° to be considered reliable for detecting clinically meaningful changes in ambulatory joint angle differences (Bian et al., 2024; Berner et al., 2020; Nüesch et al., 2017).

2. Joint angle estimation should be feasible on resource-limited wearable devices; thus, the memory requirements and computational complexity of the solutions should be minimized. A practical efficiency threshold of ≤ 20 KB was adopted, considering that typical embedded platforms used in IMU-based gait systems (e.g., STM32 or ESP32 microcontrollers) provide only about 100–512 KB of RAM. This constraint ensures the model can be deployed entirely on-device without external memory access, while maintaining flexibility for future scaling.

#### 2.4.2 Statistics

To evaluate the statistical significance of performance differences between OrientationNN and the baseline models, we conducted an independent samples t-test. Before performing the t-test, we assessed the normality of the performance metrics to ensure that the data met the assumption of a normal distribution. For each model (MLP, CNN, LSTM, Transformer, and OrientationNN), we trained 10 separate instances using optimised architectures and hyperparameters. The mean test performance across 10 participants was recorded for each training instance. This resulted in 10 independent performance values per model (N = 10), which were then compared. We used MATLAB’s t-test function to conduct a two-tailed independent samples t-test (α=0.05) to compare the performance of OrientationNN against each baseline model. The null hypothesis stated that there was no significant difference between OrientationNN and the baseline models.

## 3 Results

### 3.1 OrientationNN vs. OpenSense

**FIGURE 3:**
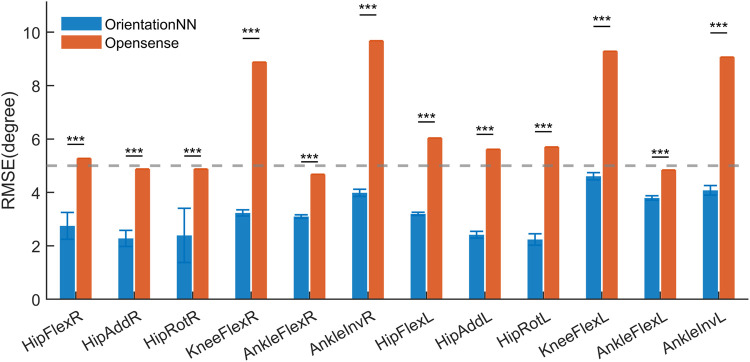
Comparison of average RMSE between OrientationNN and OpenSense across 12 lower limb joint angles.

Figure 3 shows that OrientationNN achieved significantly lower RMSEs than the OpenSense inverse kinematics approach across all 12 joint kinematic variables (p < 0.001). The average RMSEs of OrientationNN were below 5°, whereas those of OpenSense ranged between 5° and 10°.

The intra-subject evaluation demonstrated that OrientationNN achieved significantly lower RMSEs across all 12 kinematic variables compared with other machine learning models (p < 0.001). Specifically, the average errors (mean ± SD) for each variable were as follows: HipFlexR (2.59° ± 0.08°), HipAddR (2.19° ± 0.15°), HipRotR (2.07° ± 0.13°), KneeFlexR (3.22° ± 0.12°), AnkleFlexR (3.11° ± 0.06°), AnkleInvR (4.01° ± 0.12°), HipFlexL (3.19° ± 0.06°), HipAddL (2.42° ± 0.13°), HipRotL (2.25° ± 0.22°), KneeFlexL (4.62° ± 0.14°), AnkleFlexL (3.79° ± 0.09°), and AnkleInvL (4.06° ± 0.18°). These results indicate that OrientationNN provides clinically meaningful accuracy (RMSE < 5°) for all lower limb joints.

### 3.2 Joint angle profiles

**FIGURE 4:**
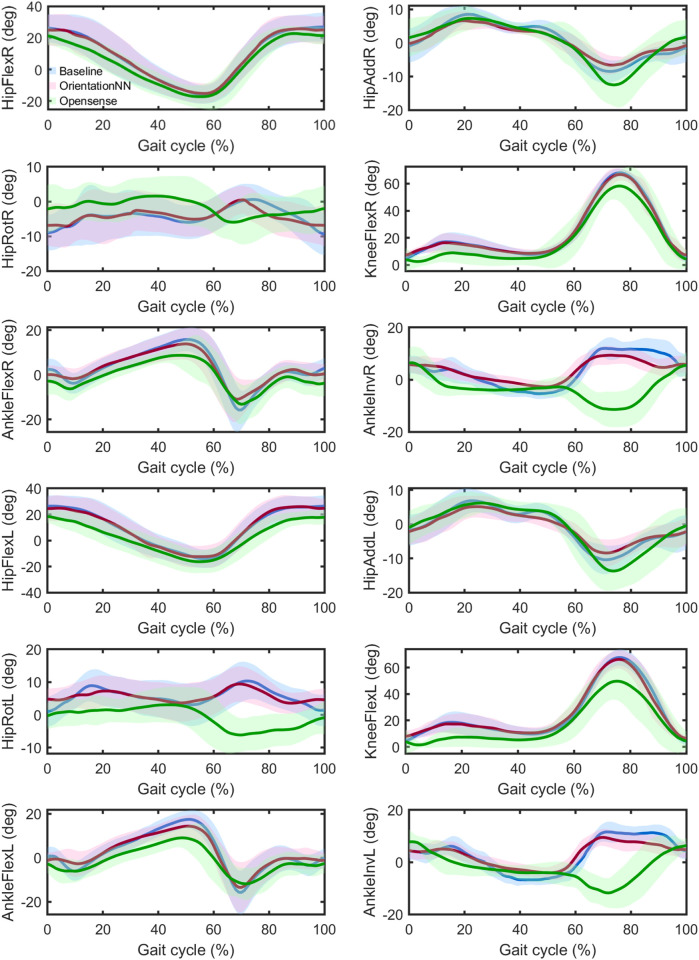
Joint angle estimation results over the whole gait cycle. Shaded regions represent the across-subject standard deviation at each normalized time point, illustrating the variability of joint-angle estimates across participants. In the time-normalized gait cycle (0%–100%), heel strike corresponds to 0% and 100%, while toe-off occurs around 50%–60%.

Figure 4 illustrates the estimated lower limb joint angles over a complete gait cycle (0%–100%) for 12 kinematic variables, including hip, knee, and ankle joints on both right and left limbs. Ground-truth trajectories obtained from optical motion capture are shown for reference (Baseline, cyan).

Across all twelve kinematic variables, the error distributions differed between the OrientationNN and OpenSense. For OrientationNN, small deviations from the baseline were mainly observed around heel strike (0%–10%) and toe-off (50%–60%), corresponding to rapid transitions in segment motion. Most local fluctuations were mild ( < 3°) and symmetrically distributed across both limbs. For OpenSense, larger deviations occurred primarily during mid-swing (70%–90%) in hip flexion and knee flexion, and near heel strike in ankle dorsiflexion and inversion. The errors reached up to 8°–10°, showing clear phase-dependent drift.

### 3.3 Model efficiency analysis

**FIGURE 5:**
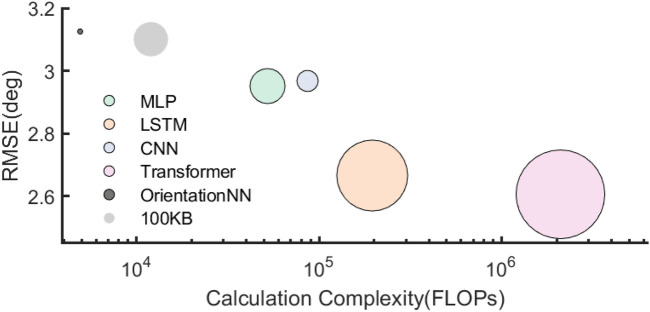
Computational efficiency of baseline models and OrientationNN.

The bubble chart (Figure 5) illustrates the trade-off between computational complexity (FLOPs) and prediction error (RMSE) for different neural network models. The bubble size represents the model parameter size (in KB). The proposed OrientationNN achieved an RMSE of 3.13 ° with only $4.9\times 10^{3}$ FLOPs and a model size of 10.8 KB, indicating the lowest computational cost among all models. In contrast, the Transformer achieved the smallest error (2.61 ° ) but required $2.1\times 10^{6}$ FLOPs and 609 KB of parameters. The LSTM model reached an RMSE of 2.67 ° with $1.96\times 10^{5}$ FLOPs and 392 KB, while MLP and CNN produced errors of 2.95 ° and 2.97 ° , respectively, at higher or comparable complexity levels.

## 4 Discussion

This work highlights a significant advancement in IMU-based joint angle estimation, delivering three main contributions. First, we proposed OrientationNN, a lightweight AI model which integrates physics information with tiny MLP, achieving clinical significance. Our results indicate its superiority over the physics model-based solution. Second, we revealed the error distribution of both proposed AI model and OpenSense model, providing insight into model design focus over the whole gait cycle. Finally, by introducing physics information into the AI model, we significantly reduced the model reliance on computational resources, enabling more cost-effective applications.

### 4.1 OrientationNN vs. OpenSense

Compared with the traditional physics-based OpenSense approach, OrientationNN demonstrated superior accuracy and stability across all joint angle channels. The experimental results showed that the average RMSEs of OrientationNN were below 5° for all 12 gait kinematic channels, significantly lower than those of OpenSense (5°–10°) with statistical significance (p < 0.001). This improvement primarily stems from the integration of orientation-based physical information into the neural network, allowing the model to capture intrinsic joint dynamics rather than relying solely on data-driven feature mapping. Clinically, achieving an RMSE below 5° indicates that OrientationNN meets the accuracy requirements for rehabilitation monitoring and gait assessment (McGinley et al., 2009).

From an algorithmic perspective, OpenSense relies on inverse kinematics optimization using inertial sensor signals, whose performance is easily affected by sensor drift, noise accumulation, and misalignment errors (McConnochie et al., 2025). In contrast, OrientationNN directly learns the mapping between IMU orientations and joint rotations in an end-to-end manner, effectively mitigating cumulative errors introduced by optimization. Furthermore, the introduction of the weighted Euler-angle loss enhances the model’s sensitivity to biomechanically critical directions (Liu and Popović, 2002), such as flexion/extension and rotation.

### 4.2 Joint kinematics profiles

The gait cycle analysis further revealed that OrientationNN achieved superior phase responsiveness in kinematic estimation. When compared with ground-truth trajectories obtained from optical motion capture, the predicted joint angle curves from OrientationNN exhibited high consistency across the entire gait cycle, with minimal phase lag in key movements such as hip flexion, abduction, and rotation. Conversely, OpenSense showed substantial deviations and fluctuations, particularly in hip rotation and ankle inversion during highly dynamic phases (McConnochie et al., 2025; Suvorkin et al., 2024).

In terms of error distribution, OrientationNN’s deviations were mainly concentrated in transition phases (0%–10% heel strike, 50%–60% toe-off), which are characterized by rapid angular acceleration and high inertial variability (Burnfield, 2010). Even in these challenging segments, local errors remained acceptable, and the distribution was symmetric between limbs, indicating robust inter-limb consistency. In contrast, OpenSense exhibited larger phase-dependent drift, with errors up to 8°–10° during mid-swing (70%–90%) for hip and knee flexion.

These results demonstrate that OrientationNN not only improves overall accuracy but also enhances the smoothness and physiological plausibility of the estimated trajectories. Such stability is crucial for clinical applications including gait abnormality detection and neurorehabilitation evaluation (Patel et al., 2012), where reliable and continuous kinematic signals are required for real-time feedback and control.

### 4.3 Model efficiency analysis

The efficiency analysis highlights the strong trade-off achieved by OrientationNN between computational complexity and predictive performance. The proposed model achieved an average RMSE of 3.13° with only $4.9\times 10^{3}$ FLOPs and a model size of 10.8 KB, which is substantially lower than other neural architectures. Although the Transformer achieved slightly lower error (2.61°) , it required over three orders of magnitude more computation ( $2.1\times 10^{6}$ FLOPs) and memory resources, making it impractical for real-time deployment on edge devices (Fedus et al., 2022).

The lightweight advantage of OrientationNN arises from its modular design: each joint is modeled by a compact MLP subnetwork combined with learnable static rotation matrices, preserving biomechanical interpretability while minimizing parameter redundancy (Han et al., 2015). Moreover, the dynamic rotation compensation module further refines non-linear rotational behavior without significantly increasing computational cost. These features make OrientationNN particularly suitable for deployment on wearable devices, rehabilitation robots (Dollar and Herr, 2008), and prosthetic systems, where low power consumption and real-time feedback are critical.

### 4.4 Limitation and future work

Although OrientationNN achieved strong performance in both accuracy and computational efficiency, several limitations remain. First, this study was conducted using treadmill walking data from healthy adults under controlled laboratory conditions. The model’s robustness under more complex scenarios, such as outdoor walking, uneven terrain (Hamacher et al., 2011), or sensor displacement, has yet to be validated (Prisco et al., 2025). Moreover, the current framework focuses solely on joint kinematics estimation, without explicitly modelling underlying joint dynamics such as torques or interaction forces (Thelen and Anderson, 2006). Although OrientationNN reduces dependence on explicit calibration by learning static rotation matrices, a minimal sensor-to-segment calibration is still required for real-time deployment, and these orientations cannot be entirely pre-trained. This study was conducted using treadmill walking data collected under controlled laboratory conditions. Although this setup allows reliable baseline evaluation, real-world variability such as sensor noise, placement changes, and environmental disturbances may affect model performance. Future work will therefore focus on validating the proposed method under more diverse conditions, including data augmentation and real-world walking scenarios, before extending it to pathological or rehabilitation applications.

Future work will focus on several significant directions. First, we plan to extend the current framework from kinematic estimation to dynamic modelling (Schwartz et al., 2008), enabling the prediction of joint moments and interaction forces directly from IMU data. Second, we aim to deploy and validate OrientationNN on real embedded and edge devices, assessing its real-time performance, energy efficiency, and latency in practical scenarios such as wearable gait monitoring and robotic assistance (Sze et al., 2017). Moreover, the framework can be integrated with more powerful machine learning models to achieve superior prediction accuracy over current baseline AI models.

## 5 Conclusion

This study presented OrientationNN, a lightweight and physics-informed neural network framework for IMU-based lower limb joint kinematics estimation. By embedding orientation-based physical constraints within a compact MLP architecture, the proposed model achieved both high estimation accuracy and biomechanical interpretability. Experimental results demonstrated that OrientationNN outperformed the traditional physics-based OpenSense method, achieving average RMSEs below 5° across twelve gait kinematic channels, thus meeting clinical relevance for gait assessment (Armand et al., 2016). Additionally, OrientationNN achieved this performance with substantially reduced computational complexity and parameter size, making it well suited for deployment on edge and wearable devices (Taylor et al., 2017).

## Data Availability

The original contributions presented in the study are included in the article/supplementary material, further inquiries can be directed to the corresponding author.
